# A randomized controlled trial comparing the effects of dapagliflozin and DPP-4 inhibitors on glucose variability and metabolic parameters in patients with type 2 diabetes mellitus on insulin

**DOI:** 10.1186/s13098-017-0255-8

**Published:** 2017-07-17

**Authors:** Hiroshi Nomoto, Hideaki Miyoshi, Hajime Sugawara, Kota Ono, Shingo Yanagiya, Mayuko Oita, Akinobu Nakamura, Tatsuya Atsumi

**Affiliations:** 1 Department of Rheumatology, Endocrinology and Nephrology, Faculty of Medicine and Graduate School of Medicine, Hokkaido University, North 15, West 7, Kita-ku, Sapporo, Hokkaido 060-8638 Japan; 2 Clinical Research and Medical Innovation Center, Hokkaido University Hospital, North 14, West 5, Kita-ku, Sapporo, Hokkaido 060-8648 Japan

**Keywords:** Blood glucose fluctuation, Dipeptidyl peptidase-4 inhibitors, Sodium–glucose co-transporter 2 inhibitors, Type 2 diabetes mellitus

## Abstract

**Background:**

Dipeptidyl peptidase-4 (DPP-4) inhibitors and sodium–glucose co-transporter 2 (SGLT2) inhibitors improve hyperglycemia, and the usefulness of co-administration of DPP-4 inhibitors and insulin therapy has been well established. However, it has been still uncertain whether combination therapy of SGLT2 inhibitors and insulin is superior to that of DPP-4 inhibitors and the latter. Therefore, we investigated the superiority of dapagliflozin on glucose fluctuation compared with DPP-4 inhibitors in patients with type 2 diabetes mellitus (T2DM) on insulin using a continuous glucose monitoring (CGM) system.

**Methods:**

In this prospective, randomized, open-label controlled trial, 36 patients with T2DM and treated with DPP-4 inhibitors and insulin therapy, were enrolled and allocated into two groups. The patients either switched their DPP-4 inhibitors to dapagliflozin 5 mg for 12 weeks, or continued their DPP-4 inhibitors for the same period. CGM analyses and metabolic markers were assessed before and after treatment periods.

**Results:**

In total, data from 29 patients were analyzed. There were no significant differences in the mean amplitude of glycemic excursions and other CGM profiles in either group after treatment. Within the dapagliflozin treatment group, significant reductions of body mass index and albuminuria, and increases of HbA1c, hemoglobin and hematocrit were observed, but improvement of albuminuria was not significant if compared with the DPP-4 continuation group.

**Conclusions:**

Combination therapy of dapagliflozin and insulin was not superior in glucose fluctuation to DPP-4 inhibitors on insulin. However, dapagliflozin may in part provide favorable effects on metabolism in patients with T2DM treated with insulin therapy.

*Trial registration* UMIN-CTR: UMIN000015033. Registered 2 September 2014

## Background

Important goals for treatment of patients with diabetes mellitus include suppression of macro- and microvascular complications and maintenance of good mortality related with these patients’ high risk for cardiovascular diseases (CVD) [1]. Recent comprehensive care to avoid cardiovascular risk factors dramatically reduced diabetic complications [2]; however, the mortality is still higher in patients with diabetes compared with that of those without [3]. Recently it has been suggested that glucose fluctuation is closely related to endothelial cell damage [4], known to be the first stage of atherosclerosis and a predictor of CVD [5]. Moreover, mean amplitude of glycemic excursions (MAGE), which is a marker of daily blood glucose variability relating to higher postprandial blood glucose or hypoglycemia, is known to be an important factor in determining the severity of coronary artery disease, independent of HbA1c. It is also an independent predictor of mortality [6]. Additionally, MAGE was also reported to correlate closely with oxidative stress in vivo [7]. To overcome the risk of CVD, it would be important to manage glycemic fluctuation.

To date, several anti-hyperglycemic agents are available, but remedies that can suppress glucose variability are limited. One of such agents, dipeptidyl peptidase-4 (DPP-4) inhibitors, has been known to exert glucose level dependent hypoglycemic action [8]. Additionally, combination therapy using insulin injection and DPP-4 inhibitors has reduced HbA1c levels and hypoglycemic events and also reduced glucose fluctuation such as the M value compared with insulin treatment alone [9, 10]. However, some sodium–glucose co-transporter 2 (SGLT2) inhibitors have been shown to be potent suppressors of cardiovascular risks [11, 12], but the effectiveness of SGLT2 inhibitors on suppressing glucose fluctuations in patients with type 2 diabetes mellitus, especially compared with DPP-4 inhibitors, has not been clarified. In this trial, we aimed to assess the effects of dapagliflozin, one of the SGLT2 inhibitors, on glucose fluctuation in patients with type 2 diabetes mellitus on insulin using a prospective, randomized parallel-group comparison study design.

## Methods

### Study population

We defined the inclusion criteria as follows: subjects with type 2 diabetes mellitus who were treated with a combination of the usual dose of DPP-4 inhibitors with any kind of insulin therapy for more than 3 months, and aged 20–80 years with an HbA1c level between 6.0 and 9.0%. Other oral hypoglycemics such as metformin, sulphonylureas, glinides, thiazolidine and alpha-glucosidase inhibitors were allowed and continued during the study period. We excluded patients if they were pregnant, had persistent elevation of serum transaminase levels (more than three times the upper limit of normal), had renal dysfunction [estimated GFR <45 mL min^−1^ (1.73 m^2^)^−1^], or had low body mass index (BMI <22 kg m^−2^).

### Protocol

This open-labelled, prospective, randomized, parallel-group comparison study was conducted in Hokkaido University Hospital. Following enrolment, all individuals were randomly assigned to a group by an independent organization at the beginning of the study to either continue their DPP-4 inhibitor treatment or change to dapagliflozin 5 mg once daily according to their age, BMI, HbA1c and eGFR levels. The participants monitored their daily glucose levels during conventional treatment for 3–5 days using a continuous glucose monitoring (CGM) system (iPro2^®^, Medtronic MiniMed, Northridge, CA, USA). After initial assessments, they were treated with prior DPP-4 inhibitors or 5 mg dapagliflozin for 12 weeks. The doses of insulin were titrated according to the Japan Diabetes Society guidelines, targeted at <130 mg dL^−1^ for average pre-meal blood glucose levels and <180 mg dL^−1^ for average 2-h postprandial glucose levels. Other medications were continued without change and all patients were encouraged to continue diet and exercise therapy during the study period. At the end of the study, glucose monitoring using CGM for 3–5 days was again performed. For biochemical analyses, fasting blood and urine samples were measured at the times of fitting the CGM system.

The primary endpoint of the study was the extent of change in MAGE [13]. Briefly, MAGE was calculated for each subject by taking the arithmetic mean of CGM values increased or decreased (from nadirs to peaks or vice versa) when both ascending and descending segments exceeded the value of one standard deviation (SD) of the CGM values for the same 24-h period. Secondary endpoints were changes in mean blood glucose, SD and hyper- or hypoglycaemia in the CGM (>180 or <70 mg dL^−1^), surrogate markers of beta-cell function and metabolic parameters. Concerning the diagnosis of diabetic nephropathy, the definition was based on the urine albumin to creatinine ratio ≥300 mg g^−1^ creatinine [14]. The subject enrolment period was from 7 September 2014 to 30 September 2016. The last subject completed the study in February 2017.

### Statistical analyses

The sample size was determined by assuming that dapagliflozin would improve MAGE by at least 60 mg dL^−1^ (SD = 50.0), based on a previous study which assessed efficacy of 5 mg day^−1^ dapagliflozin in patients with diabetes [15]. It was determined that 32 patients were needed to detect a significant difference with at least a power of 80% and statistical significance of 5%. To account for the potential loss of subjects, the sample size was set at 36 patients (18 per group). Results are expressed as mean ± SD or medians and 25–75% quartile. Differences of baseline characteristics between groups were assessed by unpaired *t* test or Mann–Whitney U test for continuous variables and Fisher’s exact test for categorical variables. The Kolmogorov–Smirnov test for normality was used to determine the appropriate statistical test for the continuous variables. Analysis was done on the full-analysis set, which was defined as all treated participants with available pre- and post-CGM assessments. For the primary analysis, the effects of dapagliflozin compared with DPP-4 inhibitors on MAGE were assessed by unpaired *t* test. Mean changes of metabolic parameters in both groups between baseline and the end of the survey were analyzed as the secondary analyses. We also employed paired *t* test or Wilcoxon signed-rank test for comparison of pre- and post-treatment. Group comparison for the differences of mean changes was performed using unpaired *t* test or Mann–Whitney U test. A *p* value <0.05 was considered statistically significant. Data were analyzed using Ekuseru-Toukei 2012 software (Social Survey Research Information, Tokyo, Japan).

## Results

### Baseline characteristics

We enrolled 36 individuals and randomly assigned all participants to two groups with 18 participants each. After enrolment, five participants did not complete the first CGM examination for the following reasons, three for consent withdrawal and one each for relocation and ineligibility to the criteria (low adherence). The remaining 31 participants received the first CGM assessment but two later dropped out. In total, 29 participants completed the CGM assessments.

Because our study was based on treatment assignment, we used the full-analysis set for our analyses. Table 1 presents the baseline characteristics of participants, comprising 12 women and 17 men with a mean age of 61.4 ± 8.7 years and mean HbA1c level of 7.3 ± 0.8%. There were no statistical differences in age, MAGE, levels of HbA1c, duration of diabetes, BMI, liver and renal functions between groups at the baseline. Only sex and urine albumin to creatinine ratio differed. However, prevalence of diabetic nephropathy did not show a significant difference. All were treated with insulin therapy with the usual dose of DPP-4 inhibitors with or without other peroral hypoglycemic agents. None of the participants discontinued dapagliflozin due to adverse events, although one case discontinued for other reasons as described previously.

**Table 1:** Clinical characteristics of the full analysis set

Variables	Dapagliflozin (n = 14)	DPP-4 inhibitors (n = 15)	*p* value
Age (years)	63.0 ± 7.7	60.0 ± 9.6	0.36
Female sex (n)	2	10	0.01
Duration of disease (years)	17.1 ± 7.9	14.8 ± 10.4	0.50
Hemoglobin A1c (%)	7.1 ± 0.8	7.4 ± 0.7	0.33
Body mass index (kg m^−2^)	26.5 ± 4.6	25.7 ± 2.5	0.61
Hemoglobin (g dL^−1^)^a^	14.1 ± 1.5	13.5 ± 1.7	0.35
Hematocrit (%)^a^	41.8 ± 4.5	40.7 ± 4.8	0.53
ALT (U L^−1^)	32 (17–45)	17 (14–22)	0.07^§^
Uric acid (mg dL^−1^)	5.6 ± 1.6	5.3 ± 1.3	0.61
Estimated GFR [mL min^−1^ (1.73 m^2^)^−1^]	68.8 (63.9–76.0)	73.7 (63.2–77.0)	0.76^§^
Diabetic nephropathy (%)^a^	7.1	28.6	0.33
Log UACR^a^	1.93 ± 0.85	1.33 ± 0.52	0.04
The total insulin dose (U)	11 (8–22)	14 (10–20)	0.54^§^
Insulin regimen (n)			
MDI/mix/basal	1/3/10	1/4/10	0.95
Diabetic medication (n)			
DPP-4i/Met/SU/TZD/αGI	14/10/3/1/3	15/13/3/2/1	0.80

Values are presented as the mean ± SD or median (range). p value of Dapagliflozin vs. DPP-4 inhibitors groups. ^§^ Mann–Whitney U test was applied to the following factors: estimated GFR, ALT, and the total insulin dose

*ALT* alanine-aminotransferase, *LDL* low-density lipoprotein, *GFR* glomerular filtration rate, *UACR* urine albumin to creatinine ratio, *MDI* multiple daily injection, *Mix* mixed insulins, *Basal* long-acting insulin, *DPP-4* dipeptidyl peptidase-4 inhibitors, *Met* metformin, *SU* sulphonylureas/glinides, *TZD* thiazolidines, *αGI* alpha-glucosidase inhibitors

^a^Data from 28 patients

### Glucose fluctuation and glycemic control

**Fig. 1:**
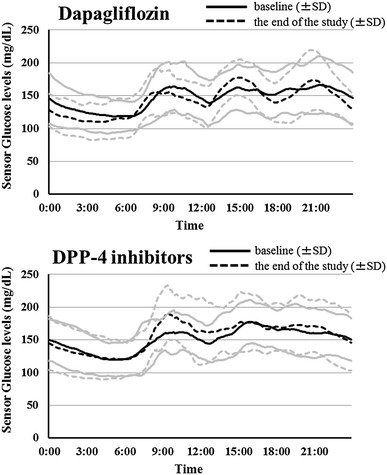
Average glucose profiles during treatment with dapagliflozin and DPP-4 inhibitors. The *black solid line* and *grey solid lines* show the mean and SD at baseline, respectively. The *black dotted line* and *grey dotted lines* show the mean and SD at the end of the study, respectively

**Fig. 2:**
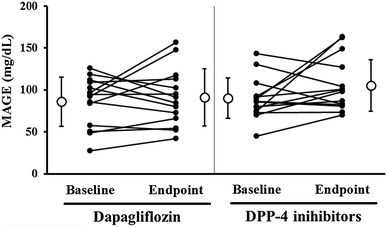
Comparison of individual changes in MAGE for each drug between the study baseline and endpoint. *White circles* and *lines* are mean and SD, respectively

As shown in Table 2 and Figs. 1, 2, the baseline CGM data were not significantly different between groups. During observation periods, MAGE was slightly increased in both groups; however, changes were not statistically significant (dapagliflozin; *p* = 0.52, DPP-4 inhibitors; *p* = 0.11). Similarly, there were no significant changes for mean blood glucose, SD of glucose levels, frequency of high or low blood glucose (>180 or <70 mg dL^−1^), and night-time hypoglycaemia between pre- and post-treatment in both groups. Concerning the surrogate markers of glycemic control, HbA1c was significantly increased only in the dapagliflozin group (*p* = 0.005), but this change did not show significant differences between the groups (*p* = 0.30) (Table 3). Throughout the study, participants maintained insulin regimens and total insulin dosages did not significantly change.

**Table 2:** Comparison of glucose variability in the two groups between baseline and endpoint

Variables	Dapagliflozin (n = 14)			DPP-4 inhibitors (n = 15)			Change difference between groups *p* value
	Baseline	Endpoint	Mean change (95% CI)	Baseline	Endpoint	Mean change (95% CI)	
MAGE (mg dL^−1^)	86.0 ± 29.4	91.0 ± 34.2	5.0 (−11.6 to 21.7)	89.8 ± 24.1	105.0 ± 30.9	15.2 (−3.8 to 34.3)	0.39
24-h mean blood glucose (mg dL^−1^)	147.4 ± 26.6	144.6 ± 22.7	−2.8 (−13.6 to 8.0)	150.5 ± 21.8	155.2 ± 26.1	4.7 (−7.1 to 16.4)	0.32
24-h SD values glucose levels	35.3 ± 11.4	39.1 ± 11.7	3.9 (−1.7 to 9.4)	37.5 ± 8.5	43.6 ± 11.7	6.1 (0.0 to 12.3)	0.56
24-h AUC > 180 (mg dL^−1^ day^−1^)	6.9 ± 6.3	6.9 ± 6.1	−0.1 (−4.0 to 3.9)	7.3 ± 6.3	11.6 ± 10.0	4.2 (0.6 to 7.8)	0.09
24-h AUC < 70 (mg dL^−1^ day^−1^)	0 (0–0)	0.1 (0–0.3)	0 (−0.5 to 1.0)	0 (0–0.1)	0 (0–0.1)	0.0 (−1.0 to 0.5)	0.21^†^
Nighttime AUC < 70 (mg dL^−1^ day^−1^)	0 (0–0)	0 (0–0.4)	0 (0 to 1.0)	0 (0–0)	0 (0–0)	0.0 (−0.2 to 1.0)	0.05^†^
The total insulin dose (U kg^−1^)	0.23 ± 0.19	0.23 ± 0.21	0.00 (−0.03 to 0.02)	0.25 ± 0.13	0.25 ± 0.13	0.00 (−0.01 to 0.01)	0.64

Baseline data are presented as the mean ± SD or median (25–75% CI). p values: mean changes from baseline to the end of the study between the Dapagliflozin group and the DPP-4 inhibitors group. ^†^ Mann–Whitney U test

*MAGE* mean amplitude of glycemic excursions, *24-h* all day, *SD* standard deviation, *AUC* area under the curve, *nighttime* 00:00 to 08:00 h

**Table 3:** Changes between baseline and endpoint in metabolic and laboratory markers in the two groups

Variables	Dapagliflozin (n = 14)	DPP-4 inhibitors (n = 15)	Change difference between groups *p* value
	Mean change (95% CI)	Mean change (95% CI)	
HbA1c (%)	0.48 (0.17 to 0.79)^††^	0.24 (−0.13 to 0.61)	0.30
Glycated albumin (%)^a^	1.04 (−0.14 to 2.21)	0.55 (−0.91 to 2.01)	0.58
Body mass index (kg m^−2^)	−0.90 (−1.42 to −0.39)^††^	0.12 (−0.12 to 0.36)	0.001
Systolic BP (mmHg)^b^	−2.8 (−11.5 to 5.8)	4.5 (−5.7 to 14.6)	0.25
Diastolic BP (mmHg)^b^	4.1 (−3.2 to 11.4)	2.0 (−2.9 to 6.9)	0.61
Hemoglobin (g dL^−1^)^a^	0.44 (0.05 to 0.83)^†^	0.01 (−0.43 to 0.47)	0.13
Hematocrit (%)^a^	1.61 (0.46 to 2.77)^†^	−0.23 (−1.61 to 1.15)	0.04
ALT (U L^−1^)	−2.0 (−26.0 to 6.0)	1.0 (−4.0 to 11.0)	0.03^‡^
Uric acid (mg dL^−1^)	−0.54 (−1.10 to 0.03)	0 (−0.37 to 0.32)	0.11
Estimated GFR [mL min^−1^ (1.73 m^2^)^−1^]	1.3 (−11.7 to 9.0)	3.0 (−4.9 to 13.6)	0.42^‡^
Log UACR^a^	−0.16 (−0.32 to 0)^†^	0.04 (−0.10 to 0.17)	0.05

Values are mean ± SD or median (25–75% CI). *P* values: mean changes from baseline to the end of the study between the Dapagliflozin group and the DPP-4 inhibitors group. ^†^ *P* < 0.05 and ^††^ *P* < 0.01 between baseline and the end of the study, (paired *t* test or Wilcoxon signed-rank test). ^‡^ Mann–Whitney U test

*BP* blood pressure, *ALT* alanine-aminotransferase, *LDL* low-density lipoprotein, *HDL* high-density lipoprotein, *GFR* glomerular filtration rate, *UACR* urine albumin to creatinine ratio

^a^Data from 28 patients

^b^Data from 27 patients

### Other metabolic parameters and relationships between MAGE

Dapagliflozin treatment significantly ameliorated BMI and albuminuria (*p* = 0.002 and 0.045, respectively) and elevated hemoglobin and hematocrit levels (*p* = 0.03 and 0.01, respectively), but not between group comparisons of albuminuria and hemoglobin (*p* = 0.052 and 0.13, respectively). Because there was a significant difference in the baseline urine albumin-to-creatinine ratio (UACR) between groups, we compared ΔUACR between groups using an analysis of covariance adjusted for the baseline UACR. This analysis also showed that there was no significant difference between ΔUACR in group comparison (p = 0.21, adjusted for baseline UACR). In addition, there was no significant correlation between baseline UACR and ΔUACR in the dapagliflozin group (r = −0.382, p = 0.18). Moreover, in comparison to the DPP-4 continuation group, liver alanine-aminotransferase elevation was significantly improved in the dapagliflozin group (*p* = 0.03) (Table 3).

With dapagliflozin treatment, eGFR was significantly higher in patients with improved MAGE compared with those without (p = 0.02) (Table 4), and the effects of dapagliflozin were partially diminished (not statistically significant) in participants with relatively impaired renal function (Table 5). No other metabolic or baseline characteristics including age, BMI, surrogate markers of glycemic control, duration of diabetes, and insulin doses correlated with improvement in MAGE.

**Table 4:** Comparison of the backgrounds of dapagliflozin group with or without amelioration of MAGE

Variables	Dapagliflozin (n = 14)		*p* value
	Amelioration (Δ MAGE < 0) (n = 6)	Deterioration (Δ MAGE ≥ 0) (n = 8)	
Baseline MAGE (mg dL^−1^)	100.2 ± 25.3	75.3 ± 29.2	0.11
Age (years)	62.7 ± 8.9	63.3 ± 7.3	0.90
BMI (kg m^−2^)	25.9 ± 4.7	26.9 ± 4.9	0.71
Duration of disease (years)	16.7 ± 4.5	17.5 ± 10.0	0.84
HbA1c (%)	7.0 ± 0.5	7.2 ± 1.0	0.65
1,5-Anhydro-d-glucitol (μg mL^−1^)	8.3 ± 4.5	8.8 ± 5.1	0.85
ALT (IU L^−1^)	42.2 ± 39.7	35.8 ± 22.6	0.73
Estimated GFR [mL min^−1^ (1.73 m^2^)^−1^]	79.7 ± 12.5	62.2 ± 11.7	0.02
The total insulin dose (U)	21.7 ± 21.3	13.1 ± 7.8	0.38

Values are mean ± SD. *p* value of amelioration vs deterioration groups

*BMI* body mass index, *ALT* alanine-aminotransferase, *GFR* glomerular filtration rate

**Table 5:** Comparison of the effects of dapagliflozin on metabolic parameters with or without renal dysfunction

Variables	Dapagliflozin (n = 14)		*p* value
	eGFR ≥ 70 [mL min^−1^ (1.73 m^2^)^−1^] (n = 6)	eGFR < 70 [mL min^−1^ (1.73 m^2^)^−1^] (n = 8)	
ΔMAGE (mg dL^−1^)	−3.4 ± 24.1	11.4 ± 31.9	0.34
ΔHbA1c (%)	0.3 ± 0.7	0.6 ± 0.4	0.35
ΔBMI (kg m^−2^)	−1.2 ± 0.4	−0.7 ± 1.1	0.31

Values are mean ± SD. *p* value of eGFR ≥ 70 vs eGFR < 70

*eGFR* estimated glomerular filtration rate, *MAGE* mean amplitude of glycemic excursions, *BMI* body mass index

## Discussion

In this trial, we aimed to verify the superiority of dapagliflozin to therapy with DPP-4 inhibitors and insulin in type 2 diabetes patients for MAGE. To date, some clinical studies have been conducted on the relationship between glycemic fluctuation and macrovascular risks. The risk factors for cardiovascular events such as endothelial cell function, oxidative stresses, and intra-media thickness were shown to be strongly correlated with glycemic variability in patients with diabetes mellitus [7, 16, 17]. There is no robust evidence that suppression of glycemic variability directly reverses cardiovascular events, but recently, some anti-diabetic remedies that can inhibit glucose fluctuation were shown to improve surrogate markers of cardiovascular risk factors [18, 19]. Therefore, it is important to moderate not only HbA1c but also glycemic fluctuation.

Previous studies have shown that both DPP-4 inhibitors and SGLT2 inhibitors affect glucose fluctuation. DPP-4 inhibitors have a potent hypoglycemic effect during the hyperglycemic state via glucose-dependent insulin secretion by increasing the active serum GLP-1 and GIP concentration, or by reducing hypoglycemia by diminishing insulin secretion and cancelling glucagon suppression under normal to hypoglycemic conditions [20]. GIP has been reported to act as a potent insulin releaser under hyperglycemic conditions. Other hand, GIP is also thought to be a physiological bifunctional blood glucose stabilizer because insulin secretion action does not occur and the glucagon response was improved under normal or hypo-glycemic conditions [21]. These glucose level-dependent mechanisms of incretins suggest that DPP-4 inhibitors can improve glycemic control and reduce glucose fluctuations in patients with type 2 diabetes mellitus compared with other insulin secretagogues [22]. Concerning the combination of the DPP-4 inhibitors with insulin therapy, meta-analysis revealed that co-administration of DPP-4 inhibitors and insulin injection achieved better glycemic control without increasing hypoglycaemia [23].

SGLT2 inhibitors also possess hypoglycemic actions and a preferable effect on glucose variability. The effects of SGLT2 inhibitors rely on the filtered load of glucose, and thus it is expected that their hypoglycemic action will work potently under hyperglycemic conditions [24]. To avoid hypoglycemia under insufficient SGLT2 conditions, compensatory SGLT1 reabsorption prevents excessive glycosuria [25] and excessive hypoglycemic action may be inhibited. In addition, increasing the plasma glucagon concentration [26] and hepatic gluconeogenesis [26], and enhancing lipolysis in patients with type 2 diabetes mellitus may also prevent hypoglycemia [27]. A randomized controlled trial using CGM comparing luseogliflozin and a placebo with type 2 diabetes mellitus found significant improvements in glucose variability and mean blood glucose with luseogliflozin [28]. Even in type 1 diabetes mellitus, dapagliflozin also reduced MAGE in a dose dependent manner [15], which provided evidence of the beta-cell independent effect of SGLT2 inhibitors on glucose fluctuation. A recent prospective study comparing the efficacy of administration of SGLT2 inhibitor with a co-administration of DPP-4 inhibitor and SGLT2 inhibitor on multiple insulin injection therapy revealed that there were no significant changes in MAGE and SD on sensor glucose measurements among insulin therapy alone, or with the addition of DPP-4 inhibitors and/or SGLT2 inhibitor. However, night-time hypoglycaemia was significantly decreased in regimen including the SGLT2 inhibitor compared with insulin monotherapy [29].

Our study showed as follows; accompanied with insulin, switching from DPP-4 inhibitors to dapagliflozin maintained an equivalent glucose fluctuation and all-day and nocturnal hypoglycaemia prevalence without significant insulin dosage change. However, we could not verify the superiority of dapagliflozin compared with DPP-4 inhibitors in MAGE, and HbA1c deteriorated only in the dapagliflozin group. As described above, the DPP-4 inhibitors’ potency in reducing glucose fluctuations has been shown. Switching to dapagliflozin did not show additional improvement on MAGE, which might be because baseline glucose variations were already suppressed in response to the DPP-4 inhibitors in insulin therapy. In addition, this result might be somewhat different if insulin titration was performed to maintain preferable glucose levels in the dapagliflozin group because generally glucose variation is influenced largely by mean blood glucose levels. That is, the higher the mean glucose levels are, the higher the glucose fluctuation will be. Even in DPP-4 continuation, glycemic control worsened in some participants. Our protocol did not allow participants to change the dosage or frequency of their DPP-4 inhibitors or other drugs taken for comorbidities in this group. Thus, deterioration of such changes observed in the DPP-4 inhibitor groups might be a result of adherence to dietary and exercise therapy. Taken together, combination therapy with SGLT2 inhibitors and insulin injection may be superior to insulin monotherapy in avoiding hypoglycaemia, and there seems to be no obvious differences between DPP-4 inhibitors and SGLT2 inhibitors as a partner of insulin in this context.

In addition, our study revealed that patients with improved MAGE after dapagliflozin administration possessed relatively preferable renal function. DPP-4 inhibitors are known to exert hypoglycemic action even with renal dysfunction [30]. On the other hand, the hypoglycemic effects of SGLT2 inhibitors rely on proper renal function. In addition, a previous report which assessed the efficacy of luseogliflozin on individuals with different renal function using CGM revealed that patients with renal insufficiency showed not only attenuated glucose lowering effects but also a lack of SD improvement in the blood glucose [31]. Taken together, the effect on glucose fluctuation of SGLT2 inhibitors may require relatively good renal function.

Both DPP-4 inhibitors and SGLT2 inhibitors have been reported to have some pleiotropic effects on metabolisms other than the hypoglycemic action. Previous reports verified that incretin agents including DPP-4 inhibitors exert renal protective effects in vitro and in vivo against increased renal oxidative stress under hyperglycemia [32]. The pooled analysis of prospective clinical trials using linagliptin resulted in significant improvement in albuminuria in patients with type 2 diabetes mellitus and renal dysfunction independent of changes in blood glucose and blood pressure [33]. Our study showed that albuminuria improved only in the dapagliflozin group after switching from DPP-4 inhibitors, but not between group comparison in the absence of a change in glucose fluctuation and blood pressure. The impact of SGLT2 inhibitors on renal protection has recently attracted much attention. In the diabetic state, the tubuloglomerular feedback system is known to break down and to result in causing glomerular hyper-filtration. It has been revealed that SGLT2 inhibitors reduced the reabsorption of glucose in the proximal tubule resulting in normalized tubuloglomerular feedback and glomerular hyper-filtration [34]. Although there are only a few large-scale clinical trials that have focused on the impact of SGLT2 inhibitors on renal protection, one of the SGLT2 inhibitors, empagliflozin, was reported to be associated with slower progression of kidney disease and lower rates of clinically relevant renal events in patients with type 2 diabetes mellitus [35].

Our data also showed significant elevation of hemoglobin and hematocrit with dapagliflozin treatment. These phenomena were thought to be due to up-regulation of erythropoietin rather than dehydration as previously reported [36], and such hematopoietic action might in part exert favorable effects on diabetic kidneys [37].

Finally, dapagliflozin significantly reduced BMI and liver enzyme as expected. Previously, we investigated the effects of ipragliflozin on body composition in Japanese patients with type 2 diabetes mellitus and revealed that body weight loss accompanied with improvement of liver dysfunction was driven mainly by reducing visceral fat mass and reducing water volume [38]. Even with co-administration of SGLT2 inhibitors and insulin injection, a recent meta-analysis reported that this combination resulted in significantly lower HbA1c, body weight and insulin dosage compared with placebo [39]. Considering that DPP-4 inhibitors do not affect body weight and insulin therapy tends to increase it, switching from DPP-4 inhibitors to dapagliflozin might possess merits from this point of view.

The strengths of our study are as follows: this was the first randomized controlled trial directly comparing DPP-4 inhibitors and an SGLT2 inhibitor on glucose fluctuation in insulin therapy; there were no significant biases in the pre-treatment for type 2 diabetes mellitus in both study arms; and we could assess the relationship between glucose fluctuations and other metabolic parameters. However, our small sample size may limit our ability to draw a conclusion. Other major limitations of this study were a lack of double blinding, the short study duration, and a lack of dietary uniformity because of the ambulatory care setting. In addition, some participants withdrew their consent after study inclusion and randomization. To resolve these potential issues, our findings need to be validated with a larger scaled, long term, dietary-controlled double-blind trial.

## Conclusions

In conclusion, combination therapy with dapagliflozin and insulin injection did not show glucose fluctuation superiority over DPP-4 inhibitors on insulin therapy. Although potential pleomorphic effects of dapagliflozin on metabolism might exist, further investigation would be necessary in future.

## Abbreviations

CVD: cardiovascular diseases
DPP-4: dipeptidyl peptidase-4
SGLT2: sodium–glucose co-transporter 2
BMI: body mass index
CGM: continuous glucose monitoring
MAGE: mean amplitude of glycemic excursions
SD: standard deviation

## References

[1] Sarwar N, Gao P, Seshasai SR (2010). Diabetes mellitus, fasting blood glucose concentration, and risk of vascular disease: a collaborative meta-analysis of 102 prospective studies. Lancet.

[2] Gregg EW, Li Y, Wang J (2014). Changes in diabetes-related complications in the United States, 1990–2010. N Engl J Med.

[3] Lind M, Garcia-Rodriguez LA, Booth GL (2013). Mortality trends in patients with and without diabetes in Ontario, Canada and the UK from 1996 to 2009: a population-based study. Diabetologia.

[4] Risso A, Mercuri F, Quagliaro L (2001). Intermittent high glucose enhances apoptosis in human umbilical vein endothelial cells in culture. Am J Physiol Endocrinol Metab.

[5] Fathi R, Haluska B, Isbel N (2004). The relative importance of vascular structure and function in predicting cardiovascular events. J Am Coll Cardiol.

[6] Su G, Mi SH, Li Z (2013). Prognostic value of early in-hospital glycemic excursion in elderly patients with acute myocardial infarction. Cardiovasc Diabetol.

[7] Monnier L, Mas E, Ginet C (2006). Activation of oxidative stress by acute glucose fluctuations compared with sustained chronic hyperglycemia in patients with type 2 diabetes. JAMA.

[8] Ahren B, Schmitz O (2004). GLP-1 receptor agonists and DPP-4 inhibitors in the treatment of type 2 diabetes. Horm Metab Res.

[9] Hong ES, Khang AR, Yoon JW (2012). Comparison between sitagliptin as add-on therapy to insulin and insulin dose-increase therapy in uncontrolled Korean type 2 diabetes: CSI study. Diabetes Obes Metab.

[10] Takahara M, Shiraiwa T, Kaneto H (2012). Efficacy of sitagliptin on blood glucose fluctuation in Japanese type 2 diabetic patients with basal-supported oral therapy. Endocr J.

[11] Wu JH, Foote C, Blomster J (2016). Effects of sodium–glucose cotransporter-2 inhibitors on cardiovascular events, death, and major safety outcomes in adults with type 2 diabetes: a systematic review and meta-analysis. Lancet Diabetes Endocrinol.

[12] Neal B, Perkovic V, Mahaffey KW (2017). Canagliflozin and cardiovascular and renal events in type 2 diabetes. N Engl J Med.

[13] Service FJ, Molnar GD, Rosevear JW (1970). Mean amplitude of glycemic excursions, a measure of diabetic instability. Diabetes.

[14] Haneda M, Utsunomiya K, Koya D (2015). A new classification of diabetic nephropathy 2014: a report from joint committee on diabetic nephropathy. J Diabetes Investig.

[15] Henry RR, Rosenstock J, Edelman S (2015). Exploring the potential of the SGLT2 inhibitor dapagliflozin in type 1 diabetes: a randomized, double-blind, placebo-controlled pilot study. Diabetes Care.

[16] Ceriello A, Esposito K, Piconi L (2008). Oscillating glucose is more deleterious to endothelial function and oxidative stress than mean glucose in normal and type 2 diabetic patients. Diabetes.

[17] Hu Y, Liu W, Huang R (2010). Postchallenge plasma glucose excursions, carotid intima-media thickness, and risk factors for atherosclerosis in Chinese population with type 2 diabetes. Atherosclerosis.

[18] Mita T, Katakami N, Shiraiwa T (2016). Sitagliptin attenuates the progression of carotid intima-media thickening in insulin-treated patients with type 2 diabetes: the sitagliptin preventive study of intima-media thickness evaluation (SPIKE): a randomized controlled trial. Diabetes Care.

[19] Esposito K, Giugliano D, Nappo F (2004). Regression of carotid atherosclerosis by control of postprandial hyperglycemia in type 2 diabetes mellitus. Circulation.

[20] Nauck MA, Kleine N, Orskov C (1993). Normalization of fasting hyperglycaemia by exogenous glucagon-like peptide 1 (7-36 amide) in type 2 (non-insulin-dependent) diabetic patients. Diabetologia.

[21] Christensen M, Vedtofte L, Holst JJ (2011). Glucose-dependent insulinotropic polypeptide: a bifunctional glucose-dependent regulator of glucagon and insulin secretion in humans. Diabetes.

[22] Park SE, Lee BW, Kim JH (2017). Effect of gemigliptin on glycaemic variability in patients with type 2 diabetes (STABLE study). Diabetes Obes Metab.

[23] Chen C, Yu Q, Zhang S (2015). Assessing the efficacy and safety of combined DPP-4 inhibitor and insulin treatment in patients with type 2 diabetes: a meta-analysis. Int J Clin Exp Pathol.

[24] Ferrannini E, Veltkamp SA, Smulders RA (2013). Renal glucose handling: impact of chronic kidney disease and sodium–glucose cotransporter 2 inhibition in patients with type 2 diabetes. Diabetes Care.

[25] Rieg T, Masuda T, Gerasimova M (2014). Increase in SGLT1-mediated transport explains renal glucose reabsorption during genetic and pharmacological SGLT2 inhibition in euglycemia. Am J Physiol Renal Physiol.

[26] Merovci A, Solis-Herrera C, Daniele G (2014). Dapagliflozin improves muscle insulin sensitivity but enhances endogenous glucose production. J Clin Investig.

[27] Ferrannini E, Muscelli E, Frascerra S (2014). Metabolic response to sodium–glucose cotransporter 2 inhibition in type 2 diabetic patients. J Clin Investig.

[28] Nishimura R, Osonoi T, Kanada S (2015). Effects of luseogliflozin, a sodium–glucose co-transporter 2 inhibitor, on 24-h glucose variability assessed by continuous glucose monitoring in Japanese patients with type 2 diabetes mellitus: a randomized, double-blind, placebo-controlled, crossover study. Diabetes Obes Metab.

[29] Okajima F, Nagamine T, Nakamura Y (2017). Preventive effect of ipragliflozin on nocturnal hypoglycemia in patients with type 2 diabetes treated with basal-bolus insulin therapy: An open-label, single-center, parallel, randomized control study. J Diabetes Investig.

[30] Chen M, Liu Y, Jin J (2016). The efficacy and safety of dipeptidyl peptidase-4 inhibitors for treatment of type 2 diabetes mellitus patients with severe renal impairment: a meta-analysis. Ren Fail.

[31] Jinnouchi H, Nozaki K, Watase H (2016). Impact of reduced renal function on the glucose-lowering effects of luseogliflozin, a selective SGLT2 inhibitor, assessed by continuous glucose monitoring in japanese patients with type 2 diabetes mellitus. Adv Ther.

[32] Fujita H, Morii T, Fujishima H (2014). The protective roles of GLP-1R signaling in diabetic nephropathy: possible mechanism and therapeutic potential. Kidney Int.

[33] Groop PH, Cooper ME, Perkovic V (2013). Linagliptin lowers albuminuria on top of recommended standard treatment in patients with type 2 diabetes and renal dysfunction. Diabetes Care.

[34] Thomson SC, Rieg T, Miracle C (2012). Acute and chronic effects of SGLT2 blockade on glomerular and tubular function in the early diabetic rat. Am J Physiol Regul Integr Comp Physiol.

[35] Wanner C, Inzucchi SE, Lachin JM (2016). Empagliflozin and progression of kidney disease in type 2 diabetes. N Engl J Med.

[36] Lambers Heerspink HJ, de Zeeuw D, Wie L (2013). Dapagliflozin a glucose-regulating drug with diuretic properties in subjects with type 2 diabetes. Diabetes Obes Metab.

[37] Sano M, Takei M, Shiraishi Y (2016). Increased hematocrit during sodium–glucose cotransporter 2 inhibitor therapy indicates recovery of tubulointerstitial function in diabetic kidneys. J Clin Med Res.

[38] Yamamoto C, Miyoshi H, Ono K (2016). Ipragliflozin effectively reduced visceral fat in Japanese patients with type 2 diabetes under adequate diet therapy. Endocr J.

[39] Tang H, Cui W, Li D (2017). Sodium–glucose co-transporter 2 inhibitors in addition to insulin therapy for management of type 2 diabetes mellitus: a meta-analysis of randomized controlled trials. Diabetes Obes Metab.

